# The Application of Evidence-Based Medicine in Individualized Medicine

**DOI:** 10.3390/biomedicines11071793

**Published:** 2023-06-23

**Authors:** Peter Van de Vliet, Tobias Sprenger, Linde F. C. Kampers, Jennifer Makalowski, Volker Schirrmacher, Wilfried Stücker, Stefaan W. Van Gool

**Affiliations:** Immune-Oncological Centre Cologne (IOZK), D-50674 Cologne, Germany

**Keywords:** evidence-based medicine, real-world data, immune-oncology, individualized treatment, clinical evidence, health-related quality of life, randomized controlled trials

## Abstract

The fundamental aim of healthcare is to improve overall health of the population by providing state-of-the-art healthcare for individuals at an affordable cost. The foundation for this system is largely referred to as “evidence-based medicine”. Too often, evidence-based medicine is based solely on so-called “best research evidence”, collected through randomized controlled trials while disregarding clinical expertise and patient expectations. As healthcare gravitates towards personalized and individualized medicine, such external clinical (research) evidence can inform, but never replace, individual clinical expertise. This applies in particular to orphan diseases, for which clinical trials are methodologically particularly problematic, and evidence derived from them is often questionable. Evidence-based medicine constitutes a complex process to allow doctors and patients to select the best possible solutions for each individual based on rapidly developing new therapeutic directions. This requires a revisit of the foundations of evidence-based medicine. A proposition as to how to manage evidence-based data in individualized immune-oncology is presented here.

## 1. Introduction

Cancer is the second leading cause of death, accounting for about 1 in 6 human deaths. Due to this population burden, intensive preclinical and clinical research is aimed at finding solutions. Immunotherapy in cancer has become a hot topic for basic science, translational and clinical research, largely in the aftermath of awarding Nobel Prizes for Medicine first in 2011 to Ralph Steinman for his discovery of dendritic cells (DCs) and their role in adaptive immunity, and to Bruce Beutler and Jules Hoffmann for their discoveries concerning innate immunity activation; and in 2018 to James Allison and Tasuku Honjo for their discovery of checkpoint inhibitors as immunomodulatory cancer therapy through inhibition of negative immune regulation. Despite these advances, a cure for cancer has not yet been found.

In translational immune-oncology, insights derived from multiple domains of immunotherapy are translated rapidly into clinical applications for cancer patients. This is realized through the development of a multi-phase combined treatment strategy against cancer, including individualized multimodal immunotherapy with different immunotherapy technologies during and after standard of care anticancer treatment [1]:

1. Strengthening of the standard of care anticancer treatments with immunogenic cell death (ICD) immunotherapy using oncolytic viruses (e.g., Newcastle Disease Virus) in combination with local modulated electrohyperthermia to induce in situ ICD of tumor cells, aimed at immune modulation in the tumor micro-environment plus conditioning of the patient’s immune system before vaccination.
2. Active specific immunotherapy after completion of chemotherapy, aimed to induce a strong specific T cell immune response against tumor-associated antigens, which is realized through the production of an individualized IO-VAC^®^ DC vaccine (DE_NW_04_MIA_2015_0033 and DE_NW_04_MIA_2020_0017 since 27 May 2015; IOZK GmbH, Cologne, Germany). This vaccine is an approved advanced therapy medicinal product (ATMP) that consists of autologous mature DCs loaded with autologous tumor antigens and matured with danger signals (in this case, a Newcastle Disease Virus with cytokines cocktail). The vaccine is unique for each patient due to the use of autologous DCs and tumor antigens. During this phase of treatment, individualized modulatory immunotherapies are implemented.
3. Maintenance of anticancer tumor control and expansion of immune-covered antigenic spectrum through repetitive 5-day ICD immunotherapy cycles, which target and kill potential newly developed tumor cells; and through vaccination with more universal long-peptide vaccines like WT1 and/or survivin.
4. Immunization through a booster IO-VAC^®^ vaccine, at least 6 months after the second IO-VAC^®^ (DE_NW_04_MIA_2015_0033 and DE_NW_04_MIA_2020_0017 since 27 May 2015; IOZK GmbH, Cologne, Germany), to increase immune response and induce a memory response.

Distinct from “personalized” medicine (meaning: tumor-specific treatment), “individualized medicine” should also take into account the uniqueness of (the immune system of) each individual patient. The term “individualized multimodal immunotherapy” (IMI) was coined in 2020 [2] and indicates the application of a combination of different immunotherapy modalities. This includes application of a combination of unique patient-derived biologic materials (like tumor antigens, dendritic cells, expanded T cells), but also allows for necessary treatment adaptation based on the unique patient-specific immune response. Individualized multimodal immunotherapy is aimed to partake in each step of an anticancer multiphase combined treatment strategy [1].

The German legislation framework of the “individueller Heilversuch” (individualized treatment) [3] allows for this forward, state-of-the-art type of clinical treatment of patients as described above. Within this framework, patient and doctor together discuss all possible treatment options, design a mutually agreed-upon treatment plan, and solidify this with an informed consent and treatment contract. This allows for highly individualized treatment, maximally benefitting each individual patient. The effectiveness and value of such individualized treatment was assessed through retrospective analyses of 50 adults with primary glioblastoma multiforme, for which an improved overall survival compared to published data was found [4]. In addition, such interventions cause profoundly fewer side effects than other systemic therapies [5], and they are more cost-efficient, which is especially valuable when considering costs of newly approved cancer drugs are reportedly set at USD > 100,000 per patient per year [6].

Notwithstanding these achievements, analyses, results and arguments, representatives of academic mainstream medicine maintain reservations about this type of individualized medicine, and thus facilitate rejections of cost-coverage or reimbursement by health insurance companies. To exemplify, the following arguments for declining IO-VAC^®^ treatment reimbursement were given by a German health insurance company (case 11966448):

- This treatment is an experimental application;
- Concrete studies on the combination of the chosen methods are missing;
- Efficacy can only be demonstrated through controlled clinical trials with a large number of patients. Therefore, treatment with new DC vaccines should be carried out exclusively in trials until efficacy is proven. The basic rule here is that these studies should take place in centers, and participation must be voluntary and free of charge as this is an experimental therapy in which the benefits and risks are still unknown.

We advocate for additional decision-making pathways for early identification of effective drugs and/or treatments alongside randomized-controlled trials (RCTs), as recommended by Roussos Torres and Epstein [7]. In this paper, we outline the restrictions of a decision-making process based solely on RCTs, offer solutions without compromising the fundamental principles of evidence-based medicine, and illustrate how it can benefit not only future but current patients.

## 2. Evidence-Based Medicine

Healthcare fundamentally aims to improve overall population well-being by providing state-of-the-art solutions for individuals at an affordable cost. The foundation for this system is referred to as “evidence-based medicine” (EBM), which is defined as “the conscientious, explicit, judicious and reasonable use of modern, ‘best’ evidence in making decisions about the care of individual patients“ [8]. EBM integrates clinical experience and patient values with the “best” available research information. In theory, this means integrating individual clinical expertise and patient values with the most recent and robust available external clinical evidence from systematic research. The external clinical evidence is then used to inform, but never replace, individual clinical expertise. In effect, it is the clinical expertise that decides whether the external evidence applies to the individual patient, and if so, how it can be integrated into a clinical decision [9]. As such, EBM constitutes a complex process to allow doctors and patients to select the best possible solutions for each individual patient.

In practice, however, EBM is limited to the implementation of best research evidence by regulators and practitioners [10]. “Best” here is exclusively understood as “evidence generated through RCTs”, and thus disregards real-world data (RWD) and clinical experience.

EBM is crucial for progress in oncology, including patient safety and high-quality treatment. This raises the question of how to generate appropriate evidence in line with the foundations of EBM. In that regard, we discuss the three cornerstones of an EBM triangle, featuring knowledge translation for the benefit of the individual patient: (1) best research evidence, (2) clinical evidence and (3) patient values. Not considering the complementarity of the three components implies certain risks:

- Disregarding patient values leads to hasty or routine-protocol medicine;
- Not considering best research evidence leads to application of empirical treatments; and
- Ignoring own clinical expertise leads to patient exposure to non-suitable treatments.

**Figure 1:**
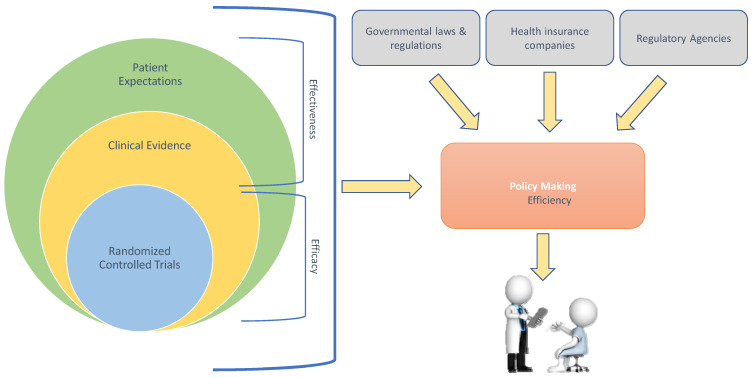
Patient-centered evidence-generation in personalized and individualized medicine aligned with the foundations of evidence-based medicine.

We briefly touch on how these aspects serve experimental treatment translation into policy making (Figure 1).

### 2.1. Best Research Evidence

The Oxford Centre for EBM—Levels of Evidence are typically referred to as a tool to assess whether a clinical intervention meets scientific evidence (external clinical evidence from systematic research). The levels range from 5 (expert opinion without critical appraisal, based on physiology, bench research or “first principles”) to 1 (systematic review of RCTs) [11]. In the latter, a new experimental arm is typically assessed against the most successful or well-established current treatment as the control arm, in combination with careful monitoring of (long-term) side effects.

The term “standard of care” was originally intended to define a minimum level of care considered acceptable without committing malpractice. Over time, the term has evolved into the “appropriate” or “best” care, a level of care that balances risk and benefit, outcomes and costs, and legal fears, and that is based on scientific evidence. Because the RCT, and especially the systematic review of several RCTs, is commonly accepted as the highest level of scientific evidence, it has become the “gold standard” for judging whether a treatment does more good than harm. With this justification, regulators and insurers are (too) often restricting reimbursements of treatment costs to data retrieved solely from RCT [12,13], as presented in the case in the Introduction. As a result, the other levels of evidence are mostly disregarded. By doing so, (1) one ignores what constitutes best research evidence; (2) one denies individual clinical expertise that does eventually decide whether the external evidence applies to the individual patient; (3) shortcomings and/or restrictions of RCTs are overlooked; and (4) cost and time required to develop new therapies are needlessly driven up [14]. This has become subject to debate and questioning, not at least by critical assessment of the power and attitude of big pharma companies [15]. This is why reflection on decisive evidence in addition to and beyond RCT methodology (in immune-oncology) is crucial, particularly considering individualized immune-oncology.

1. Traditional RCTs focus on hypothesis testing by comparing an experimental arm (e.g., therapeutic intervention) to a control arm (no intervention). For the concept to work as intended, the administration of the experimental treatment should be the sole difference between the experimental and the control group. By nature, these are study designs, not treatment designs. The primary end of the trial is not to treat patients, but rather to generate generalizable medical knowledge. Aside from the financial and logistical complications (such trials take years to design and run, time these patients simply do not have), it implies that half of the patients that might benefit from a novel intervention are denied such a benefit, as they do not meet scientific design criteria. Of the patients who are accepted, half will be placed in the control group, again not receiving treatment. This conflicts with the right of the patient to consent to individualized treatment decisions based on to their condition. It is argued that patients explicitly consent to this when they endorse trial participation, but, in almost all countries, patients often have no other options left outside palliative treatment or euthanasia. This is a pertinent ethical concern, given that the Helsinki Declaration requires that “the well-being of the individual research subject must take precedence over all other interests” [16,17].
2. Traditional clinical trials produce “average” results for a given outcome variable and cannot answer questions related to why therapies work in certain situations but not in others. Even the most rigorous and clearly reported RCT cannot predict if a given intervention will be effective in any specific individual. Ironically, these questions are of most value to patients, and thus of most interest to clinicians. This is especially valid in (immune-)oncological research, where the importance of evaluating patient-unique genetic and molecular hallmarks to design and administer situationally the least harmful and most effective treatment (tailored treatment), gains momentum out of apparent necessity [18,19].
3. Traditional clinical trials require multiple clear and strict eligibility criteria to ensure that the study population is similar in all baseline factors that may affect the potential benefits and risks from the intervention studied. This not only requires large study populations, but also implies that patients considered at greater risk of adverse events from the trial or not expected to benefit will be excluded, as well as patients with comorbid conditions or receiving concurrent therapies. In addition, patients who are not expected to live through a clinical study, arguably those in greatest need, cannot be included. While these criteria make sense to eliminate bias and balance for unknown covariates in such experimental setup, it introduces its own “optimized patient”-bias. Furthermore, it is argued that overly strict eligibility criteria result in lower patient accrual, which is already a challenge for rare or orphan diseases where the sample sizes are small and overall survival is low. This problem is clearly presented in recent work by Liau et al. [20], who had to modify their trial design testing an autologous tumor lysate-loaded dendritic cell vaccine for treatment of glioblastoma, for feasibility and/or ethical reasons. Patients who need it most cannot benefit from an experimental treatment, even though these are the patients who will receive the treatment in clinical reality. West [21] argues that for patients who cannot await results from RCTs, which often take years to become available, retrospective clinical data may provide assurance instead. These pertinent issues in immune-oncology research result in study populations that are unrepresentative of the actual clinical population of patients with cancer, disregarding concerns and complications which occur in real-life treatment, while simultaneously limiting patient access to new treatments [6,22,23,24,25].
4. Traditional clinical trials do not consider rapid advances in tumor biology, which slices and dices cancer into ever smaller subsets. Indeed, it is assumed that all randomized individuals are, and will remain, homogeneous, and that no change within the set of investigated subjects occurs during the study period except the changes due to treatment. This is not true for cancers, which are known to evolve through continuous and rapid accumulation of genomic mutations. This calls for smaller, shorter, focused approaches, as targeted therapies will, by nature, become more individualized [14,26,27]. This complexity of personalized medicine means that a model subset cannot accurately present patient heterogeneity.
5. Rapid expansion of novel immune-oncology agents may result in a need to compare new therapies and new combination treatments against each other and/or against an expanding list of standard care treatments. Conducting multiple RCTs to facilitate these comparisons gets overly complicated, expensive and resource-intensive. Due to the complicated and time-expensive setup of RCTs, they simply cannot keep up with current technological advancements, which partially explains why only 1 in 5000 to 100,000 new therapeutic inventions make it to market application [14,28].
6. As RCTs are study designs in support of, but not creating, EBM, it is important that “all” evidence should be publicly available, both published and unpublished, to avoid evidence-biased medicine. Transparency in reporting is essential; if methods and data are not shared in an unbiased and open format, it contributes to the so-called reproducibility crisis [29]. It is commonly understood that about 50% of research is not published, with a strong bias towards positive results in published data (publication and reporting bias) [30,31]. Furthermore, a sample review suggested that 45% of the industry-funded trials were not required to report any results, as opposed to 6% of trials funded by the National Institutes of Health and 9% of the trials that were funded by other government or academic institutions [32]. This is of concern, given that sponsorship of drug and device studies by manufacturing companies leads to more favorable efficacy results than sponsorship by other sources (industry/sponsorship bias) [33,34].

### 2.2. Clinical Evidence

The impact of healthcare interventions can be assessed in different ways. Efficacy is the extent to which an intervention does more good than harm under ideal circumstances (“Can it work?”). Effectiveness describes whether an intervention does more good than harm when provided under usual circumstances of healthcare practice (“Does it work?”). Efficiency defines the effect of an intervention in relation to the resources it consumes (“Is it worth it?”) [35]. RCTs largely focus on efficacy, but the effectiveness of an intervention equally depends on provider compliance, patient adherence and ultimately costs, and therefore can and should not be ignored. As Haynes [35] stated: “We need more effectiveness studies to sort the fool’s gold from the true gold, and efficiency studies to tell us if the price of extraction is a bargain.” The emergence of an effectiveness perspective reflects a paradigm shift towards societal impacts of treatment, including financial burden, and toward a thorough reexamination of what is considered relevant scientific evidence to evaluate treatments. Effectiveness studies emphasize flexible, innovative methods for delivering treatments that are more applicable to usual care, and generally include larger populations that are more representative in terms of medical complexity and thus better fitted to evaluate long-term clinical outcomes.

According to Ioannidis [36], the usefulness of clinical research requires (1) the existence of a real problem to address; (2) proper context placement; (3) sufficient information gain; (4) patient-centeredness; (5) pragmatism; (6) reasonable value-for-money; (7) non-futility; and (8) transparency. Very few clinical studies meet even six of these eight criteria. Moreover, it is far more common for clinicians to overestimate than to underestimate benefits, but to underestimate rather than overestimate harm [37]. Particular attention should be drawn to the use of clinical guidelines, which are produced by professional associations and written by people with financial ties to interested companies, thus potentially driving overuse and overdiagnosis [34,38].

With increasing frequency, clinicians encounter situations without relevant evidence from basic or applied research toward a particular case or therapy; or situations where they face difficulties applying an ever-increasing amount of highly diverse evidence to the care of individual patients. In such cases, patients are the best source of valid, reliable, high-quality data on the severity, impact and duration of symptoms and side effects, and therefore should be consistently included as clinical evidence [39]. Unquestionably, this requires the acquisition and development of new skills for physicians. This includes the regular critical appraisal of recent literature while safeguarding patient values and expectations, in their decision-making and delivery of “appropriate” care [40]. Although the value of RCTs is not questioned, the controlled settings in which RCTs are conducted do not necessarily allow for extrapolation of the findings to the heterogeneous patient population treated in routine clinical practice.

The advancement of immune-oncology therapy has transformed the treatment landscape for numerous solid tumors. Emergence of novel agents harnessing the host immune system is expected to continue on an upward trajectory for the foreseeable future. This is even more prominently the case in individualized treatment. Subsequently, real-world data (RWD) have gained the interest of different stakeholders in cancer care. RWD are data relating to patient health status and/or the delivery of health care, collected from a variety of sources other than traditional clinical trials [41]. In this way, RWD are a solution for situations where a robust clinical trial is not practicable because of, for example, low recruitment prospects, prohibitive anticipated costs, resource needs and/or ethical prohibition [10,24,42]. In such cases, RWD provide a valuable and rich data source beyond the confines of traditional epidemiological studies, clinical trials and lab-based experiments, with lower costs in data collection compared to the latter [43]. This principle has recently successfully been applied to the effectiveness of anti-COVID-19 strategies [43]. This principle equally applies to clinics and institutions that specialize in personalized and individualized treatment with high innovative potential [5,7]. To effectively treat patients in daily clinical practice and to be able to give patients realistic treatment expectations requires estimation of the real-world effectiveness of therapies on the basis of (individual) patients’ characteristics. If used and analyzed appropriately, RWD represent a rich source of valid and unbiased evidence, comparable to controlled trials (so-called “regulatory grade RWD”) [10].

In this constantly evolving environment, developers and regulators confront unique questions and drive critical developments and regulatory processes that could be informed by RWD. In recent years, different initiatives were started by regulators, to improve the use of such data in the regulatory process, and to initiate a shift from “policy by direction” to “policy by precedence”. This would allow industry to draw on the merits of previous submissions, rather than being specifically directed by the regulatory bodies to provide the information they require [10]. Additionally, as data can be structurally collected and so questions can be answered in shorter periods compared to RCTs, it is furthermore likely this will reduce the costs of extensive and time-consuming clinical trials.

### 2.3. Patient Expectations

The principal subject of EBM is the patient. Decision-making should maximize the patient’s individual health. Such informed decision-making should be based on: (1) description of the nature of the decision; (2) discussion of alternatives; (3) discussion of risks and benefits; (4) discussion of related uncertainties; (5) assessment of the patient’s understanding; and (6) elicitation of the patient’s preference. Informed decision making should be guided by patients’ preferences for a treatment, which often differ from those of clinicians. These elements are not always met and, in combination with unreliable medical evidence and a tsunami of misleading reports in the media, risk leading to suboptimal, nonpatient-centered decision-making [40]. However, the ultimate stakeholder in medical decision making must be the patient. Patients are increasingly assuming more responsibility in their treatment decisions, and their knowledge and expectations are of considerable importance. Knowledge about and expectations of treatment are likely to affect patients’ decisions in dealing with daily issues during treatment, and may not only affect quality of life [44], but equally increase accrual to novel therapy, trials and therapy. When patients discuss treatment with their oncologists, they want to know “How does the drug work in people like me?”, “How will I feel?”, and “How will this impact my life?” [39]. In the field of immune-oncology, treatment advances have produced major progress in terms of both survival and quality of care, with patients’ quality of life becoming a more prominent objective. Patients value moments, milestones, and quality of life. The absence of (severe) side effects with cancer vaccines in immunotherapy [5] plays a significant role toward patient happiness. Increased survival therefore requires a particular focus on monitoring and safeguarding patient-reported outcomes, such as health-related quality of life (HRQoL).

HRQoL is defined as “a patient’s general subjective perception of the effect of illness and intervention on physical, psychological and social aspects of daily life” [45]. Including HRQoL in clinical or epidemiological studies and in clinical practice therefore assists in optimizing expectations: (1) HRQoL facilitates an understanding of the patient’s perspectives on loss or gain as a result of a disease or a medical intervention; (2) HRQoL offers insight into the balance between therapeutic benefit and adverse effects of an intervention as experienced by the patient; and (3) HRQoL aids in defining responses in addition to or in the absence of quantifiable endpoints such as tumor regression [46,47,48]. HRQoL complements neurological examination and evaluation of cognitive function by a patient-centered self-report approach. Evidence suggests that treatment side effects were the sole factor to negatively affect patients’ quality of daily life during treatment. These where outweighed by the benefits of having a support object that makes patients feel good, the subjective perception of the efficacy of the anticancer treatment, and the positive effects of good supporting relationships including with the treating physician, which were listed as positive contributions to quality of life [49]. Ensuring that patients are adequately prepared to make decisions requires professional assistance to explore both the treatment options and the medical evidence, so that the potential outcomes that matter most to the patient can be accurately determined. This process is crucial in EBM, and safeguards the capacity of an individual to make free choices [50].

In summary, the current trial landscape inadequately reflects the population; generalizability of trial results into clinical practice carries considerable uncertainty; and the huge costs and time-expense of RCTs cannot be sustained and even hamper innovation in immune-oncology. An additional argument is the hard fact that the “standard of care” oncology treatments are associated with over 90% mortality at two years for some metastatic cancers, despite a multitude of clinical trials [51], reflecting the desperate need for new intervention therapies. Evidential standards conflict with classical RCTs [16], but some questions about therapy do not require randomized trials, e.g., successful interventions for otherwise fatal conditions. When the life-expectancy is shorter than the actual trial, the next best external evidence should be applied, as suggested by West [21]. RWD provides an alternative source of reliable evidence besides RCTs, and both should be supplemented with HRQoL to maintain focus on the patient. This especially applies in immune-oncology, where unmet medical needs are greatest, life expectancy lowest and population burden highest. Coincidentally, clinical trials for orphan drugs take nearly twice as long as trials for non-orphan drugs, even though response to therapy, albeit somewhat distinct from other cancer treatments, is characterized by durable tumor suppression and associated long-term survival [7,52,53]. Orphan cancer entities, like cervical cancer or malignant glioma in particular, are often accompanied by an exceeded year-life-loss (YLL). Since industry does not invest much money in research here, treatment options are fewer than for more widespread cancer entities such as breast, colon or prostate cancer. For that reason, Sackett et al. (1996) stated that EBM should not be restricted to RCTs and meta-analysis, but instead should involve tracking down the best external evidence to answer any particular clinical question, or as they stated: “Evidence-based medicine is not ‘cookbook’ medicine” [18]. This requires a shift from the question “Is the therapy of overall benefit to the group” to “who can benefit?” [51].

## 3. Moving Forward: Managing Evidence-Based Data in Personalized and Individualized Medicine

Individualization of treatment, made necessary by the reality that no two tumors are alike, no two patients are alike, no clinical course is identical, and tumor–host interactions are highly individual, is more and more often adopted in immune-oncology [2,7,14,54]. We have entered a new era, with novel insights leading to new, individualized, more effective treatment options with higher success rates. This includes opportunities for advanced malignancies, which improve HRQoL, are less toxic, are tailored to specific patient and disease characteristics, and are potentially less expensive. Considering the limitations of RCTs, it is time that medical organizations, regulators and health insurances expand their views and support new types of clinical studies and so-called individualized precision-oncology “to offer the right drug for the right patient at the right time” [55]. This would be a true reflection of the fundamentals of EBM as defined by Sackett [8] and Sackett et al. [18].

Lead authorities such as the US Department of Health and Human Services—Food and Drug Administration (US FDA, responsible for all approvals of clinical studies in the US) and the EU (European Medicines Agency [EMA]/EU Policy Department for Economic, Scientific and Quality of Life Policies) have recognized this need, and have called for a review of the current decision-making process on the basis of clinical trials. They recommended that data collected in a non-RCT setting and that well-designed retrospective studies should be considered in future decision making [41,56,57,58]. Such data collected at an individual level not only provide critical evidence, but also improve randomization and evaluation of efficiency required by the modern world of (immune-)oncology.

RWD has an unleashed potential, as have N-of-1 cancer trials. Both are fundamentally different from the classical RCT approach, in that they are patient-centered, where the drugs are fitted to the patient, rather than drug-centered, where the patient is fitted to the drug trial [17,23,59]. The voluminosity and complexity of RWD calls for development of more appropriate, sophisticated, and innovative data processing and analysis techniques, while maintaining scientific rigor in the gathering and analysis of research findings, and attention to data ethics to harness its power [43,60]. This “regulatory grading” of RWD to ensure available evidence of acceptable quality is recognized by authorities such as the US FDA and the EMA. For instance, the EMA and the EU initiated the Adaptive Pathways program to allow for RWD to support regulatory submissions for drug approval and dramatically accelerate such approval. Most recently, the Canadian Real-world Evidence for Value of Cancer Drugs (CanREValue) collaboration developed a rating tool to generate and use real-world evidence to support drug funding decisions [61]. These programs address novel study designs, end points, methodologies and statistical approaches, alongside alignment in communication between industry and regulators. Such programs should incorporate recommendations for further research in personalized medicine as presented recently by Fosse et al. [30], who identified five critical points of focus: (1) clinically relevant translational research; (2) robust model development; (3) transparency and education; (4) revised regulation; and (5) interaction with clinical research and patient engagement. In 2011, the Oxford Centre for EBM classified N-of-1 trials as Level 1 evidence useful for treatment decision-making in individual patients [17,23]. N-of-1 trials are studies used to test the effects of treatment in a cross-over setting within one patient, with the aggregation of N-of-1 trials for the evaluation of new interventions being considered as an alternative study design to traditional RCTs for such situations where patient recruitment is a challenge [62]. Reporting guidelines are provided for the application in medical sciences [63], which are based on shared decision-making between patients and practitioners, thereby bringing EBM into real clinical practice. Here as well, authorities are in support of novel techniques, as stated by the EMA: “In very rare diseases, the combined evaluation of single case studies may be the only way to provide evidence. Also combined analysis of individual case reports or observational studies should be considered” [64]. The narrative is definitively evolving.

## 4. Conclusions

Personalized and individualized medicine reflect the reality that no two tumors are alike, no two patients are alike, and no clinical course is identical, not even within a group of seemingly similar patients. This is due to numerous clinical variations related to host or environment-dependent factors, which translate to a necessity for individualized multimodal treatment as part of multi-phase combined treatment strategies against cancer. Individualized multimodal treatment requires a careful review of the classical principles of hierarchy of evidence and a “back-to-the-roots”-reflection on what constitutes EBM. It is critical to keep in mind that the autonomy of the individual patient should always be at the forefront (Figure 1).

Active involvement of all relevant stakeholders (researchers, clinicians, industry, regulators and patients) is a prerequisite for success, so that we will be able to effectuate a change toward improved translation of individualized medicine. Each extra day of life with good quality of life in cancer patients is invaluable in itself.

## Data Availability

Data availability is not applicable.
